# Impact of rapid blood culture identification PCR panel on optimal antibiotic use in methicillin-susceptible *Staphylococcus aureus* bacteremia

**DOI:** 10.1128/spectrum.00629-24

**Published:** 2024-10-22

**Authors:** Jahnavi Yetukuri, Dimple Patel, Aiman Bandali, Pamela Giordano, Robert Roland, Jason Kessler

**Affiliations:** 1 Ernest Mario School of Pharmacy, Rutgers University, Piscataway, New Jersey, USA; 2 Department of Pharmacy, Morristown Medical Center, Morristown, New Jersey, USA; 3 Department of Pharmacy, Overlook Medical Center, Summit, New Jersey, USA; 4 Department of Medicine, Overlook Medical Center, Summit, New Jersey, USA; 5 Department of Medicine, Morristown Medical Center, Morristown, New Jersey, USA; NHLS Tygerberg/Stellenbosch University, Cape Town, Western Cape, South Africa

**Keywords:** rapid diagnostic tests, bloodstream infections, blood culture, *Staphylococcus aureus*

## Abstract

Rapid identification of methicillin-susceptible *Staphylococcus aureus* (MSSA) bacteremia may optimize antibiotic use and clinical outcomes. The study objective was to assess the impact of the BioFire® blood culture identification (BCID) polymerase chain reaction (PCR) panel on antibiotic use and clinical outcomes in patients with MSSA bacteremia. This was a retrospective chart review of adult inpatients with MSSA bacteremia during the pre-PCR (June 2018–December 2019) and post-PCR (June 2020–December 2021) implementation periods. The primary endpoint was the difference between the pre-PCR and post-PCR groups in time to optimal MSSA antibiotic therapy (oxacillin or cefazolin). Secondary endpoints included duration of anti-MRSA antibiotic use, in-hospital mortality, hospital and ICU lengths of stay, duration of bacteremia, and 30-day MSSA-related and all-cause readmissions. Two hundred patients were included in the study (100 per group). Median time to optimal therapy was reduced by 19.9 hours in the post-PCR group (49 vs 29.1 hours, respectively, *P* < 0.001). PCR implementation was associated with significant reductions in the initiation of empiric anti-MRSA antibiotic therapy (98% vs 84%, *P* = 0.001), duration of empiric anti-MRSA antibiotic use (44.2 vs 20.9 hours, *P* < 0.001), and duration of bacteremia (68.6 vs 47.3 hours, *P* = 0.015). No significant differences in length of stay, in-hospital mortality, or 30-day MSSA-related or all-cause readmissions were observed. In patients with MSSA bacteremia, BCID PCR panel implementation decreased time to optimal MSSA antibiotic therapy, duration of bacteremia, and duration of empiric anti-MRSA antibiotic therapy.

**IMPORTANCE:**

In this retrospective study of 200 patients with methicillin-susceptible *Staphylococcus aureus* (MSSA) bacteremia, the implementation of the BioFire® blood culture identification polymerase chain reaction (PCR) panel was associated with a decreased time to optimal MSSA antibiotic therapy and shorter durations of empiric anti-MRSA antibiotic therapy and bacteremia. The findings demonstrate the significant role of rapid PCR testing and routine stewardship review in optimizing antimicrobial therapy and management of MSSA bacteremia.

## INTRODUCTION

*Staphylococcus aureus* bacteremia is associated with high rates of morbidity and mortality (1). Delay in appropriate antibiotics can lead to increased mortality risk, prolonged hospitalizations, and metastatic complications, including endocarditis, osteomyelitis, meningitis, and septic emboli (1). The optimal antibiotic therapies for methicillin-susceptible (MSSA) and methicillin-resistant (MRSA) *S. aureus* differ significantly. Optimal antibiotic choices for MSSA bacteremia are nafcillin and oxacillin. Cefazolin is considered an acceptable alternative due to similar 30-day mortality and readmission to nafcillin and oxacillin (2). Vancomycin is the preferred antibiotic for MRSA bacteremia and is often used empirically for *S. aureus* infections. Vancomycin, however, is considered to be an inferior choice for the treatment of MSSA bacteremia due to its association with prolonged length of stay, increased risk of mortality, and persistent bacteremia (3). Additionally, treatment with other beta-lactam antibiotics, such as piperacillin-tazobactam and ceftriaxone, has been associated with suboptimal outcomes in MSSA bacteremia, such as higher rates of treatment failure and 30-day mortality (2, 4). Thus, the most appropriate antibiotic choices for MSSA bacteremia are nafcillin, oxacillin, and cefazolin. Ideally, patients with MSSA bacteremia should be initiated on one of these agents shortly after pathogen identification in order to minimize the risks of mortality and complications.

Following the detection of growth in blood culture bottles and initial Gram staining, traditional culture methods require up to 24 hours for pathogen identification and 48 hours for susceptibility results, at which point antibiotic therapy can be adjusted to target MSSA or MRSA. Penicillin-binding protein 2a (PBP2a) testing may be used to reduce the time to differentiation of MSSA and MRSA. PBP2a testing, however, can only be conducted after *S. aureus* is identified by traditional culture methods. The 2016 Infectious Disease Society of America guidelines on antibiotic stewardship recommend implementing rapid diagnostic testing along with conventional culture methods on blood specimens in order to reduce time to initiating appropriate antibiotic therapy (5–7). The BioFire® rapid blood culture identification (BCID) polymerase chain reaction (PCR) panel identifies *S. aureus* and methicillin resistance genes, including *mecA/C* and mec right-extremity junction (MREJ), within 2 hours of detection of growth in the blood culture bottle, which is markedly shorter than results with traditional methods or PBP2a testing.

Previous retrospective studies of rapid PCR diagnostic testing in patients with *S. aureus* and coagulase-negative staphylococci bacteremia resulted in significantly decreased time to optimal antibiotic therapy and numerical reductions in length of stay and mortality (8, 9). These studies included small subgroups of patients with MSSA bacteremia. Outcomes with rapid diagnostic testing in larger populations with MSSA bacteremia remain largely unclear. The purpose of this study was to evaluate the impact of the BCID PCR panel implementation on antibiotic use and clinical outcomes in patients with MSSA bacteremia.

## MATERIALS AND METHODS

This multicenter, retrospective chart review was conducted at Morristown Medical Center and Overlook Medical Center, two community teaching hospitals in northern New Jersey, USA. Patients 18 years and older with MSSA bacteremia admitted at the time of the Gram stain result were identified using the microbiology laboratory database. Those with positive blood cultures between June 2018 and December 2019 (“pre-PCR” implementation group) and between June 2020 and December 2021 (“post-PCR” implementation group) were screened. Patients were excluded if they never achieved optimal antibiotic therapy, had polymicrobial infection, had positive blood cultures in the prior 90 days, underwent penicillin-skin testing or beta-lactam desensitization, or were transitioned to comfort care or deceased within 24 hours of the Gram stain. Post-PCR group patients were additionally excluded if they did not have PCR testing performed, largely due to a shortage of the BCID panel during a portion of the study period. In addition, complicated bacteremia incidence was documented and defined as the presence of implanted prostheses, metastatic sites of infection, endocarditis, continued fever within 72 hours of initiating optimal therapy, or repeat positive blood cultures taken 2–4 days after the initial set (10).

The primary endpoint was the difference in time to optimal MSSA antibiotic therapy, defined as oxacillin or cefazolin, between the pre-PCR and post-PCR groups. Secondary endpoints included differences in duration of empiric anti-MRSA antibiotic use, duration of bacteremia, in-hospital mortality, hospital and ICU lengths of stay, and 30-day MSSA-related and all-cause readmissions. Duration of bacteremia was defined as the difference in time from the collection of the first positive blood culture to the collection of the first negative blood culture. Reasons for readmission were classified as MSSA-related based on documentation in the patient chart and clinical judgment from study investigators reviewing the chart.

**Fig 1:**
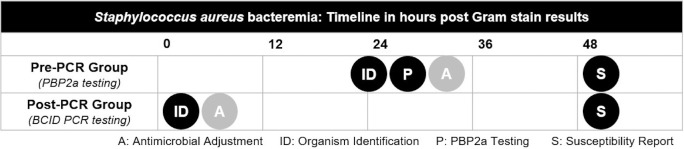
Illustration of laboratory processes and general timeline of results after positive Gram stain results. (PBP2a: Penicillin-binding protein 2a).

At the two institutions in which this study was conducted, blood culture identification methods primarily consisted of traditional culture-based methods and PBP2a testing in the pre-PCR group (Fig. 1). Following BCID implementation, PCR testing was performed 24 hours a day, 7 days per week. Infectious diseases clinical pharmacists routinely conducted antimicrobial stewardship reviews of positive blood cultures and BCID PCR results during an 8-hour time period during weekdays. Providers were subsequently contacted to adjust antimicrobial therapy as appropriate. By institutional protocol, a positive blood culture Gram stain result was considered a critical result, requiring notification to the nursing unit and provider. However, providers and pharmacists were not directly notified of each resulting BCID PCR test and, as a result, real-time stewardship interventions did not occur.

Patients with confirmed growth of MSSA in a blood specimen were identified using the microbiology laboratory database. Power analysis was conducted based on previous study findings (8). In order to meet 80% power to demonstrate a difference of 12 hours in time to optimal MSSA antibiotic therapy between groups, a sample size of at least 140 patients (70 per group) was required. Continuous data were analyzed using the Mann-Whitney *U* test, and categorical data were assessed using the two-proportion *Z*-test or Fisher’s exact test. Statistical analysis was conducted using Minitab. The significance level for alpha was 0.05.

## RESULTS

**Fig 2:**
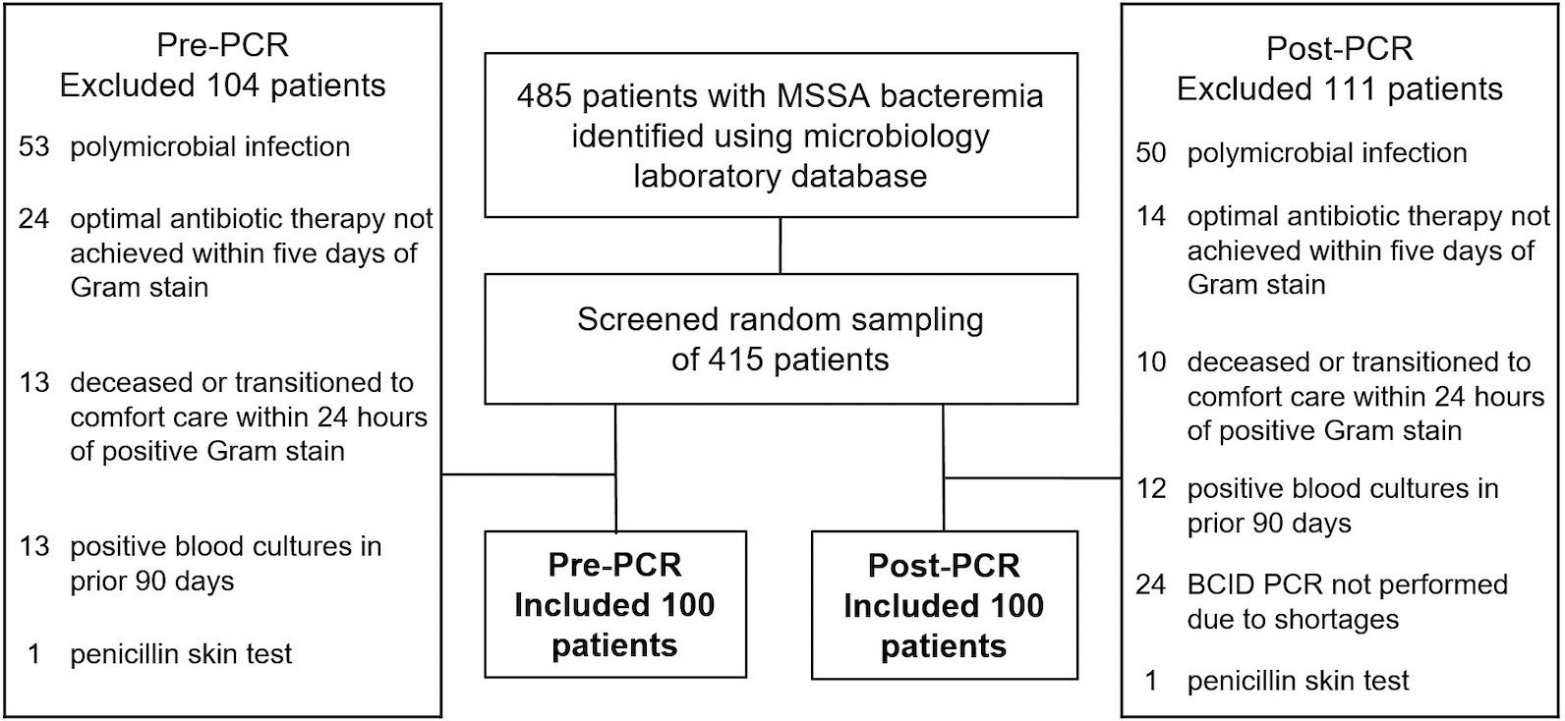
Flowchart of patients screened for study enrollment and reasons for exclusion.

Of the 485 patients identified with MSSA bacteremia during the study periods, a random sampling was screened for exclusion criteria until 100 patients per group were included (Fig. 2). Of the 415 patients screened, 215 were excluded, 104 from the pre-PCR group and 111 from the post-PCR group. The most common reasons for exclusion in both groups were polymicrobial infection or not achieving optimal antibiotic therapy within 5 days of the Gram stain. There were no discrepancies identified between BCID and final susceptibility results in terms of MSSA and MRSA.

A total of 200 patients were included in the study, with 100 patients in each group. Both groups had similar patient characteristics, including age, sex, documented beta-lactam allergies, median Pitt bacteremia scores, and ICU admission rates (Table 1). All patients included in the study were managed in consultation with an infectious diseases specialist. Significantly more patients in the pre-PCR group had complicated bacteremia compared to post-PCR group patients (73% vs 60%, *P* = 0.049). The most common types of infections in the pre-PCR group were catheter-related (26%), musculoskeletal (27%), and skin and soft tissue (23%). In comparison, the post-PCR group patients more commonly had catheter-related (30%), skin and soft tissue (24%), and endocarditis or intravascular (23%) infections. Differences in types of infection between groups were not statistically significant.

**TABLE 1:** Characteristics of patients included in the study^*d*^

	Pre-PCR (*n* = 100)	Post-PCR (*n* = 100)	*P*-value
Age (years), mean (SD)	66.1 (14)	63.8 (17.8)	0.312
Male (%)	74	71	0.635
Documented beta-lactam allergy (%)	12	12	>0.999
Pitt bacteremia score, median (IQR)	0 (0, 2)	0 (0, 2)	0.887
ID consult (%)	100	100	>0.999
ICU admission (%)	25	21	0.501
Complicated bacteremia^*a*^ (%)	73	60	0.049
Type of infection^*b*^ (%)			
Catheter-related	26	30	0.528
Musculoskeletal	27	18	0.125
Skin and soft tissue	23	24	0.868
Endocarditis/intravascular	17	23	0.287
Pneumonia	11	11	>0.999
Central nervous system/spinal	13	7	0.155
Others^*c*^	5	8	0.568

^*a*^ Complicated bacteremia defined as the presence of implanted prostheses, metastatic sites of infection, endocarditis, continued fever within 72 hours of initiating optimal therapy, or repeat positive blood cultures taken 2–4 days after the initial set.

^*b*^ Patients may have had multiple types of infection.

^*c*^ Other types of infection include intra-abdominal, urinary tract, and unknown.

^*d*^ SD, standard deviation; IQR, interquartile range.

Vancomycin was the most commonly used empiric anti-MRSA antibiotic therapy with more patients in the pre-PCR group receiving vancomycin compared to the post-PCR group (93% vs 79%, *P* = 0.004) (Table 2). Empiric anti-MRSA therapy was withheld in more patients in the post-PCR group compared to the pre-PCR group (16% vs 2%, respectively, *P* = 0.001). The most common optimal MSSA antibiotic therapy used was cefazolin. Both groups had similar median durations of optimal MSSA antibiotic therapy and use of adjunct antistaphylococcal antibiotics. In addition, there was no difference in overall source control procedures between groups. More patients in the pre-PCR group underwent surgical interventions other than catheter and foreign material removal (31% vs 19%, *P* = 0.038).

**TABLE 2:** Characteristics of antimicrobial therapies and interventions that study patients underwent during admission

	Pre-PCR (*n* = 100)	Post-PCR (*n* = 100)	*P*-value
Choice of empiric anti-MRSA antibiotic (%)			
Vancomycin	93	79	0.004
Other (daptomycin, linezolid)	5	5	
None	2	16	0.001
Choice of optimal MSSA antibiotic (%)			
Cefazolin	84	77	0.21
Oxacillin	16	23	
Duration of optimal MSSA antibiotic (days), median (IQR)	28 (28, 42)	28 (28, 42)	0.749
Adjunct antistaphylococcal antibiotic^*a*^ (%)	12	12	>0.999
Source control procedure^*b*^ (%)	54	52	0.671
Catheter removal	24	29	0.422
Foreign material removal	4	3	>0.999
Other surgical interventions	31	19	0.038

^*a*^ Adjunct antistaphylococcal antibiotic therapy includes gentamicin and/or rifamycin antibiotic.

^*b*^ Patients may have undergone multiple source control procedures.

^*c*^ MRSA: methicillin-resistant *Staphylococcus aureus*; MSSA: methicillin-susceptible *S. aureus*.

In the post-PCR group, the median time to optimal therapy was reduced by 19.9 hours (49 vs 29.1 hours, *P* < 0.001) (Table 3). The median duration of empiric anti-MRSA antibiotic therapy was significantly shorter in the post-PCR group with a reduction of 23.3 hours (44.2 vs 20.9 hours, *P* < 0.001). In addition, the median duration of bacteremia was reduced by 21.3 hours in the post-PCR group (68.6 vs 47.3 hours, *P* = 0.015). There were no significant differences in length of stay, in-hospital mortality, or 30-day MSSA-related or all-cause readmissions.

**TABLE 3:** Primary and secondary endpoints^*a*^

	Pre-PCR (*n* = 100)	Post-PCR (*n* = 100)	*P*-value
Time to optimal MSSA antibiotic therapy (hours)	49 (40.8, 67.5)	29.1 (22.3, 45)	<0.001
Duration of empiric anti-MRSA antibiotic (hours)	44.2 (34.9, 60.4)	20.9 (7.1, 31.8)	<0.001
Duration of bacteremia (hours)	68.6 (45.6, 110.1)	47.3 (35.5, 91.5)	0.015
Hospital length of stay (days)	9.4 (6, 16)	11 (7,15)	0.428
ICU length of stay (days)	3 (2, 7.5)	4 (2, 8)	0.489
In-hospital mortality (%)	12	10	0.651
30-day all-cause readmission (%)	24	27	0.626
MSSA-related	7	4	0.537

^*a*^ Data presented as median (IQR) unless otherwise specified.

## DISCUSSION

In patients with MSSA bacteremia, BCID PCR panel implementation significantly decreased the time to optimal MSSA antibiotic therapy and reduced durations of bacteremia and empiric anti-MRSA antibiotic therapy, including vancomycin. In addition, BCID PCR testing was associated with less frequent initiation of empiric anti-MRSA antibiotic therapy.

Both groups had similar patient characteristics with the exception of complicated bacteremia incidence, which was noted more frequently in the pre-PCR group. The shorter time to optimal MSSA antibiotic therapy associated with BCID PCR testing may have contributed to reduced progression to complicated bacteremia in the post-PCR group. However, the causality between BCID PCR implementation and progression to worsening infection remains uncertain. In addition, more pre-PCR group patients underwent surgical procedures other than catheter and foreign material removal, which may correlate with the increased incidence of complicated bacteremia in this group.

Several previous studies have evaluated the role of PCR testing in *S. aureus* bacteremia. Bauer and colleagues (9) assessed clinical outcomes associated with the use of rapid PCR testing and real-time notifications to an infectious diseases pharmacist in patients with *S. aureus* bacteremia. Of the 156 patients included, 75 patients had MSSA bacteremia. In this subset, time to optimal MSSA antibiotic therapy was significantly reduced by 38.4 hours (*P* = 0.002), suggesting that PCR testing and real-time pharmacist-driven interventions led to faster optimization of antimicrobial therapy. This suggests that real-time interventions with PCR testing lead to a greater reduction in time to optimal MSSA antibiotic therapy than routine stewardship. While Bauer and colleagues (9) utilized a protocol with real-time interventions, the current study validates that a reduction in time to optimal antibiotic therapy is maintained with PCR testing and routine stewardship interventions rather than real-time notifications, which is beneficial for institutions with limited resources.

In contrast to previous findings, Frye and colleagues (1) did not find a difference in time to optimal MSSA antibiotic therapy following PCR implementation in their subgroup of 77 patients with MSSA bacteremia. This result was attributed to the use of PBP2a testing in the pre-PCR group. The results of the current study, however, contradict these findings. In this study, in which PBP2a testing was utilized for the pre-PCR group, there was a significant reduction in time to optimal therapy by 19.9 hours with BCID PCR testing (*P* < 0.001). Of note, Frye and colleagues (1) considered broad-spectrum cephalosporins and beta-lactam-beta-lactamase inhibitor antibiotics as optimal therapy, which may have impacted study findings as these broad-spectrum antimicrobials are commonly used as empiric therapy. Another notable difference is the lack of antimicrobial stewardship interventions in Frye and colleagues (1). In comparison, the current study utilized routine antimicrobial stewardship review of positive blood cultures and BCID PCR results by infectious diseases clinical pharmacists, which may correlate with the significant reduction in time to optimal antibiotic therapy.

The findings of the current study add to those of Turner and colleagues (11), which demonstrated a reduced time to optimal therapy of 11.7 hours with the implementation of staphylococcal PCR testing without antimicrobial stewardship intervention for patients with MSSA bacteremia (*P* = 0.0011). In comparison, this study showed that PCR testing paired with routine antimicrobial stewardship review was associated with a 19.9-hour reduction in time to optimal therapy. This suggests that antimicrobial stewardship interventions have an additive benefit to the use of rapid diagnostic PCR testing.

In addition to time to optimal MSSA antibiotic therapy, vancomycin use was also evaluated in prior studies. Frye and colleagues (1) found a numerical reduction in the duration of vancomycin use by 8.7 hours, which was not statistically significant. In a larger retrospective study assessing the impact of PCR testing in a subgroup of 153 MSSA patients, Na and colleagues (8) demonstrated a 38.9-hour reduction in the duration of vancomycin use. In comparison, the current study resulted in a 23.3-hour reduction in the duration of empiric anti-MRSA antibiotic use. This difference in outcomes may be partially explained by the use of PBP2a testing in pre-PCR group patients, which may have lessened the impact of BCID PCR testing. In addition, patients in the post-PCR group were less frequently started on empiric anti-MRSA antibiotics. This difference between groups may be a result of the shorter time to pathogen identification with BCID PCR testing. At the two study institutions, empiric broad-spectrum anti-MRSA therapy is typically initiated when gram-positive cocci are identified in a blood sample and continued until further data are available. Due to the shorter time for MSSA identification, providers likely did not have time to react to the Gram stain result and adjusted antibiotic therapy based on the BCID results indicating MSSA.

The current study did not show significant differences in length of stay, in-hospital mortality, or 30-day readmission, which was consistent with previous findings. However, unlike prior studies, the current study also evaluated the impact of PCR testing on the duration of MSSA bacteremia, which was reduced by 21.3 hours. Although the clinical significance of this finding is unclear, prolonged *S. aureus* bacteremia has been associated with increased mortality, length of stay, and risk of metastatic complications. In a prospective observational study of 884 patients with *S. aureus* bacteremia, Minejima and colleagues (12) concluded that each additional day of bacteremia increased mortality risk by 16% compared with those at day one of bacteremia. In addition, Jarrell and colleagues (13) demonstrated a significant increase in median length of stay with persistent bacteremia, defined as 48 hours or longer (*P* = 0.0082). Based on these findings, the reduction in bacteremia duration by 21.3 hours found in the current study is likely to improve clinical outcomes but larger studies are needed.

With 200 patients included, this study met 80% power and, to our knowledge, is the largest study to date assessing the impact of rapid diagnostic PCR testing in patients with MSSA bacteremia. This is also the first study to evaluate the BioFire® rapid BCID PCR panels in this context. An evidence-based definition of optimal MSSA antibiotic therapy was used to minimize confounding variables as other antimicrobial agents have been shown to be suboptimal in comparison.

The present study has certain limitations to consider. The retrospective nature of this study renders it dependent on accurate documentation in the electronic medical record. Other assumptions made include appropriate antibiotic dosing, use of adjunct antistaphylococcal antibiotics, and initiation of source control procedures. This study has limited generalizability because patients were excluded for polymicrobial infections or not achieving optimal antibiotic therapy within 5 days of the Gram stain. On further analysis, patients most commonly did not receive optimal antibiotic therapy within 5 days of the Gram stain due to having a documented beta-lactam allergy or a suspected concomitant infection that required broader spectrum empiric antimicrobial therapy. The external validity of the study results is limited to institutions with similar protocols for BCID PCR laboratory testing and routine stewardship review. The findings of our study are most applicable to the United States population, as the prevalence of MRSA and practices for empiric therapy in other countries may vary. During the time period of the current study, MRSA prevalence at the two institutions ranged from 30% to 40%. In countries where MRSA prevalence rates are lower and empiric anti-MRSA therapies are not routinely recommended, the clinical utility of rapid diagnostic PCR testing may be different. While no significant differences were seen in mortality, readmissions, and length of stay, this study was not powered to detect differences in these outcomes.

### Conclusions

This study suggests that BCID PCR panel implementation in patients with MSSA bacteremia decreased the time to optimal MSSA antibiotic therapy, decreased the duration of bacteremia, reduced the duration of empiric anti-MRSA antibiotic therapy, and decreased the initiation of empiric anti-MRSA antibiotics. Overall, the study findings support the role of implementing rapid BCID PCR in optimizing antibiotic therapy for MSSA bacteremia. This study allowed for a real-world assessment of BCID PCR technology, including routine stewardship review, as many institutions may not have the capacity for real-time antimicrobial stewardship interventions. Future research on the impact of real-time BCID PCR interventions for MSSA bacteremia and evaluation of clinical outcomes of interest (i.e., mortality and length of stay) in adequately powered studies is warranted.
